# Editorial

**Published:** 1871-09

**Authors:** 


### Editorial.

Public Medical Library.—The members of the Chicago Medical Society are making earnest efforts to found and support a public medical library. They have already secured liberal donations and most ample and permanent accommodations in the fire-proof library building of the Young Men's Christian Association.

The library will be open daily from 9 A.M. to 10 P.M., for the public, as well as for the profession.

Will friends throughout the Northwest aid the Society in obtaining complete files of the various medical periodicals of our country, by sending stray numbers of such journals to the library.

Old catalogues of our various medical colleges are desired.

Small packages can be sent by mail; large ones, forwarded by express, will be received and charges paid by the librarian, at the rooms of the Y. M. C. A. Library.

THE LIBRARY COMMITTEE.

We call the attention of our readers to the above, and assure them that the object is a good one; and we think reliance can be placed on the success and permanence of the proposed library. Hence, let all who can, lend it a helping hand.

---

CUNDURANGO.—This, the latest pretended remedy for the cure of cancer, we have hitherto said nothing about in the *Examiner.* We said nothing because the favorable reports on the subject, including those of Dr. Bliss, of Washington, carried upon their face, or rather in their style of expression, evidence of unreliability. To prevent some of our readers from taking unnecessary trouble to procure the remedy, only to disappoint themselves and their patients, we will say that it has been under careful trial in several cases, under reliable management. and in every instance thus far has entirely failed.

---

### CIRCULAR.

---

ILLINOIS INSTITUTION FOR THE EDUCATION OF FEEBLE-MINDED CHILDREN.—This Institution, which was inaugurated in 1865, as an experimental school for the education of feeble-minded children, has been so successful in training this unfortunate class, that at the last session of the General Assembly it was organized upon an independent basis, and was incorporated as one of the permanent charitable institutions of the

State, thus completing the noble circle of public charities of the Commonwealth of Illinois.

The design and object of the Institution is to furnish the means of education to children and youth of feeble-minds, who are deprived of educational privileges elsewhere, and who are of a proper school-attending age. It is designed for those so deficent in intelligence as to be incapable of being educated at common schools, who are not epileptic, insane, or deformed.

The education furnished by the Institution will include, not only the simple elements of instruction usually taught in common schools, where that is practicable, but will embrace a course of training in the more practical matters of every-day life; the cultivation of habits of decency, propriety, self-reliance, and the development and enlargement of a capacity for useful occupation.

The combination which this Institution presents, of practical medical care, and proper physical and mental training, with efficient educational resources, will supply, it is hoped, a want which has long been felt and imperatively demanded by this unfortunate class of children and youth of the State.

The improvement and progress of the pupils have been very encouraging, and parents and friends in almost every instance have expressed satisfaction with what has been accomplished in the short time since the school was organized.

The Institution is open to the inspection of the public at all reasonable hours; and all are not only cordially invited, but are earnestly requested to visit the school.

It is a State Institution, and board and tuition are free during the school year of ten months.

It is the desire of the Trustees to ascertain accurately the number of this unfortunate class of persons in the State, and persons knowing the residence of any in Illinois will confer a favor by reporting the same to the undersigned, as it is desirable that reliable statistics may be gathered in order that proper legislation may be made in their behalf.

Those designing to apply for the admission of pupils should do so at once, as the accommodations are limited.

Applications for admission, information, etc., should be directed to

DR. C. T. WILBUR, SUPERINTEDENT,
*Jacksonville, Illinois.*

---

THE CHOLERA.—Regarding the cholera in Russia, the St. Petersburg correspondent of the London *Standard* says:

"From the first appearance of the cholera, on the 29th of August, 1870, there have been in St. Petersburg 6,817 cases, and 2,797 deaths. In Moscow and its environs the epidemic is of a very malignant character, and in some of the remote villages, where medical assistance is difficult to obtain, it has committed fearful ravages. Since the 13th of March there have been 3,568 cases in Moscow, and 1,643 deaths. The cholera has appeared in several parts of the government of Viadimir, but the proportion of fatal cases is small. At Cronstadt, up to the 29th of July, there were 439 cases and 211 deaths. At Riga, of 109 cases in two days, 78 were fatal. There have been a few cases at Mittau and also at Kovno, Vitebsk, Polotsk, and Dunaburg. At Wilna, up to the 11th of July, there had been 1,136 cases and 512 deaths. We hear of a great many people having died at Wirbellen, on the Prussian frontier. At Tamboff, a town of about 30,000 inhabitants, 2,504 cases were reported up to the 21st of July, and 1,242 deaths. At Rybinsk, a very important corn depot on the Volga, more than half the cases have proved fatal; many of the inhabitants have left the town, and business is entirely suspended. At Yaroslaff the proportion of deaths has been large, but the last accounts are more favorable."

---

THE *Independence Belge* to-day discredits the reported disappearance of cholera at Antwerp. At Konigsberg, on Wednesday, there were 127 cases and 48 deaths, and at Dantzic, on the same day, 12 cases and 10 deaths. The epidemic is decreasing in Russia.

---

THE MEDICAL REGISTER AND DIRECTORY OF THE UNITED STATES will shortly be issued by Dr. J. M. Toner, of Washington, and will include the names of 50,000 physicians. It will, moreover, contain statistics relating to all the medical schools, hospitals, medical societies and institutions of the country, and will, in this way, embrace information of value to medical men. *Boston Medical and Surgical Journal.*

---

ONE OF THE HORRORS OF WAR.—The mortality of new-born children in France during the war has been recently calculated by M. Berthillon, and the result is frightful. The author shows that for every 1000 infants under one year of age, there died in the Department of the Marne, 288; in the Department of the Oise, 295; in the Seine-et-Marne, 307; in Seine Inférieure, 318; and in Eure-et-Loire, no less than 370. This terrible mortality is, undoubtedly, one of the principal causes of the depopulation of France.—*Medical Press and Circular.*

---

CALABAR BEAN IN TETANUS.—Dr. Campbell Black, of Glasgow, has treated a case of tetanus by Calabar bean, and is convinced that the physiological action of that drug places its use in tetanus beyond the pale of empiricism.

### Mortality for the Month of July, 1871.

| | | | | | |
|---|---|---|---|---|---|
| Accidents, crushed | 1 | Delirium tremens | 3 | Measles | 12 |
| " from diving | 1 | Diphtheria | 5 | " and complications | 7 |
| " drowned | 16 | Dropsy, general | 3 | Metroperitonitis | 1 |
| " by fall | 2 | " of chest | 2 | Malformation of head | 1 |
| " kerosine | 1 | Dysentery | 33 | Old age | 8 |
| " carelessness | 1 | " chronic | 1 | Paralysis | 3 |
| " overdose morphine | 1 | Empyemia | 1 | Peritonitis | 1 |
| " by railroad cars | 7 | Entero-colitis | 10 | Phlebitis acute | 1 |
| Abcesses, illiac | 1 | Enteritis | 22 | Pneumonia | 14 |
| " lumbar | 1 | Erysipelas | 3 | " broncho | 2 |
| Anæmia | 1 | " of the face | 1 | " and complications | 2 |
| Apthae | 1 | Fever, congestive | 2 | " typhoid | 2 |
| Apoplexy | 5 | " puerperal | 5 | Polypus nasal | 1 |
| Arachnitis | 2 | " remittent | 1 | Prostration result of | |
| Aneurism of aorta | 1 | " scarlet | 6 | amputation | 1 |
| Births premature | 19 | " " & complicat's | 1 | Rheumatism | 1 |
| " still born | 55 | " malignant | 2 | " inflammatory | 1 |
| Brain, congestion of | 8 | " typhoid | 12 | Scrofula | 3 |
| " softening of | 1 | Gastromalaxia | 1 | Small-pox | 3 |
| " compression of | 1 | Gangrenous ulcer of | | Spine caries of | 1 |
| " inflammation | 7 | urinary organs | 1 | Stomach congestion of | 1 |
| " obscure disease of | 1 | Gastritis | 5 | " hemorrhage of | 1 |
| Bronchitis | 2 | Gastro-enteritis | 9 | " ulcer of | 1 |
| " chronic | 1 | Heart disease | 2 | Suicide by shooting | 1 |
| " capillary | 2 | " hypertrophy of | 1 | " by drowning | 3 |
| Cancer of cevical glands | 1 | " valvular disease of | 8 | " cutting throat | 1 |
| " of stomach | 4 | Hip-joint, disease of | 1 | " strychnine | 1 |
| Childbirth | 2 | Hernia, congenital | 1 | Syphilis | 1 |
| Chronic inflamm'n of | | Hydrocephalus | 18 | " hereditary | 2 |
| jaw bone | 1 | Inanition | 20 | Tabes mesenterica | 25 |
| Cholera infantum | 316 | Icterus | 1 | Teething | 5 |
| " morbus | 6 | Kidneys, Bright's dis- | | " complications | 10 |
| Consumption | 48 | ease of | 2 | Tumor ovarian | 1 |
| Convulsions | 105 | " disease of | 2 | Ulcer of chest | 1 |
| " puerperal | 1 | " inflammation of | 1 | Uterus rupture of | 1 |
| Croup | 6 | Liver, disease of | 1 | " inflammation of | 1 |
| Cyanosis | 1 | Lungs, congestion of | 4 | Vesical calculi | 1 |
| Cynanche maligna | 1 | " hemorrhage of | 1 | Whooping-cough | 11 |
| Cystitis, | 1 | " gangrene of | 1 | " and complications | 9 |
| Debility, general | 6 | Meningitis | 14 | Unknown | 1 |
| Diarrhœa | 49 | " cerebro-spinal | 4 | | —— |
| " chronic | 4 | " tubercular | 3 | Total | 980 |
| " and complications | 8 | | | | |

### COMPARISON.

Deaths in July, 1871, 980 | Deaths in July, 1870, 1121 | Decrease, 141

Deaths in June, 1871, 560 | Increase, 420

### AGES.

| | | | | | |
|---|---|---|---|---|---|
| Under 1 | 515 | 20 to 30 | 46 | 90 to 100 | — |
| 1 to 2 | 165 | 30 to 40 | 38 | 100 to 101 | — |
| 2 to 3 | 29 | 40 to 50 | 32 | Unknown | — |
| 3 to 4 | 22 | 50 to 60 | 24 | | —— |
| 4 to 5 | 14 | 60 to 70 | 18 | Total, | 980 |
| 5 to 10 | 29 | 70 to 80 | 11 | | |
| 10 to 20 | 32 | 80 to 90 | 5 | | |

| | | | | | |
|---|---|---|---|---|---|
| Males, | 529 | Single, | 856 | White, | 973 |
| Females, | 451 | Married | 124 | Colored, | 7 |
| Total, | 980 | Total, | 980 | Total, | 980 |

NATIVITY.

| | | | | | |
|---|---|---|---|---|---|
| Austria | | France | 3 | Poland | 1 |
| Bohemia | 10 | Germany | 60 | Russia | 1 |
| Canada | 8 | Holland | 4 | Scotland, | 2 |
| Chicago, Native | 177 | Ireland | 46 | Sweden | 8 |
| Chicago, Foreign | 531 | Italy | 0 | Wales, | 2 |
| U. S., other parts | 88 | Newfoundland | 0 | Unknown | 7 |
| Denmark | 6 | Norway | 14 | | |
| England | 11 | Nova Scotia | 1 | Total, | 980 |

MORTALITY BY WARDS FOR THE MONTH.

| Wards. | Mortality. | Pop. in 1870. | One death in |
|---|---|---|---|
| 1 | 6 | 6,531 | 1089 |
| 2 | 23 | 14,338 | 623 |
| 3 | 38 | 16,805 | 442 |
| 4 | 28 | 12,178 | 435 |
| 5 | 27 | 11,605 | 430 |
| 6 | 63 | 19,486 | 309 |
| 7 | 66 | 13,849 | 210 |
| 8 | 122 | 22,994 | 188 |
| 9 | 102 | 27,278 | 269 |
| 10 | 34 | 13,750 | 404 |
| 11 | 42 | 14,988 | 357 |
| 12 | 33 | 13,976 | 423 |
| 13 | 18 | 8,943 | 497 |
| 14 | 17 | 9,076 | 534 |
| 15 | 96 | 20,382 | 212 |
| 16 | 34 | 13,975 | 411 |
| 17 | 48 | 17,118 | 356 |
| 18 | 55 | 17,069 | 310 |
| 19 | 30 | 8,738 | 291 |
| 20 | 17 | 13,628 | 801 |

| Mortality. | |
|---|---|
| Accidents | 28 |
| County Hospital | 12 |
| Foundling Home | 12 |
| Home for Friendless | 4 |
| Hospital Alexian Brothers | 2 |
| Lake Hospital | 2 |
| Immigrants | 7 |
| Jewish Hospital | |
| Marine Hospital | |
| Mercy Hospital | 3 |
| Manslaughter | |
| Protestant Orphan Asylum | |
| St. Luke's Hospital | 1 |
| St. Joseph Orphan Asylum | 3 |
| Suicide | 6 |
| Total | 980 |

Mean Thermometer for month, —°; Rain, 0.000 inches; Deaths daily, 31⅗.

MONEY RECEIPTS FROM JUNE 20, TO JULY 22.—Dr. J. Williams, $3.00; Dr. J. B. Cory, 3.00; Dr. W. T. Knapp, 1.50; Dr. Paoli, 5.00; Dr. G. Fredighe, 3.00; Dr. S. E. Mitchell, 6.00; Dr. A. C. Buffum, 3.00; Dr. O'Donnell, 3.00; Dr. T. J. Whitten, 3.00; Dr. J. W. Duncan, 3.00; Dr. J. M. Steele, 3.00; Drs. Peck & Moore, 3.00; Dr. J. B. Newman, 3.00; Dr. J. W. Barlow, 3.00; Dr. L. Tibbets, 3.00.

BELLADONNA PLASTER.—Thomas E. Jenkins, M.D. (*Rich. & St.Louis. Med. Jour.*), says that a plaster made from the resinous extract of belladonna root is superior to that made from the spirituous extract of the leaf in the following respects: It adheres closely to the surface, requires no adhesive margins, and neither runs, exudes, nor stains the linen.

